# A Data-Driven Exploratory Analysis of the Alcohol-Harm Paradox in South-East Wales

**DOI:** 10.3390/ijerph23081058

**Published:** 2026-08-14

**Authors:** Elin H. Williams, Daniel Gartner, Paul R. Harper, Geraint I. Palmer-Liyu

**Affiliations:** 1 School of Mathematics, Cardiff University, Cardiff CF24 4AG, UK; gartnerd@cardiff.ac.uk (D.G.); harper@cardiff.ac.uk (P.R.H.); palmergi1@cardiff.ac.uk (G.I.P.-L.); 2 School of Business and Health, Aalen University, 73430 Aalen, Germany; 3 Department of Mathematics, Universitas Negeri Malang, Malang, Jawa Timur 65145, Indonesia

**Keywords:** alcohol-harm paradox, deprivation, alcohol consumption, binge drinking, alcohol-related harms

## Abstract

**Highlights:**

**Public health relevance—How does this work relate to a public health issue?**

- This research addresses the public health issue of the alcohol-harm paradox in a region where higher levels of alcohol-related harms are experienced despite having lower average annual alcohol consumption.
- Alcohol-specific deaths in the United Kingdom have seen a sharp increase following the COVID-19 pandemic.

**Public health significance—Why is this work of significance to public health?**

- Rates of alcohol-related harms differed by unhealthy behaviour (smoking, obesity, poor diet, poor exercise levels) prevalence.
- Alcohol-related venue density and proximity were explored. The venue landscape differed by deprivation, and proximity was shown to be associated with the level of alcohol-related harms.

**Public health implications—What are the key implications or messages for practitioners, policy makers and/or researchers in public health?**

- The alcohol-harm paradox is unlikely to be explained by a single-variable explanation. Rather, future research should replicate the integrated approach of this research.
- Interventions promoting an overall healthier lifestyle and more social drinking might help to mitigate the health inequalities observed.

**Abstract:**

Alcohol consumption contributes substantially to global morbidity and mortality. The alcohol-harm paradox describes the phenomenon where more deprived areas experience higher levels of alcohol-related harms despite reporting similar or lower levels of alcohol consumption compared to less deprived areas. This paradox is particularly evident in Gwent, South-East Wales, where more deprived areas experience greater levels of harms despite having lower annual alcohol consumption. This research aims to explore the impacts of various hypothesised contributors to the alcohol-harm paradox in Gwent. Publicly available cross-sectional data sources are used to explore health behaviours, demographics, alcohol outlet density, and proximity to alcohol-related venues. Factors are studied in relation to both annual alcohol consumption and binge drinking prevalence. Alcohol-related harms are considered in terms of alcohol-specific deaths and alcohol-attributable hospital admissions. The findings reinforce the known complexity of the alcohol-harm paradox, and suggest that it is unlikely to be attributable to a single factor. Elevated prevalence of unhealthy behaviours in more deprived areas and proximity to alcohol venues traditionally associated with cheaper alcohol emerged as potential influential factors. These exploratory findings highlight the importance of targeted public health messaging addressing an overall healthier lifestyle and less harmful consumption behaviours, to reduce alcohol-related health inequalities.

## 1. Introduction

Alcohol consumption places a significant burden on disease and mortality. Alcohol consumption was associated with 2.6 million deaths worldwide in 2019 and is associated with more than 200 diseases, injuries, and health conditions [1]. In the UK, the number of alcohol-specific deaths has increased 38% since 2019 [2], and it is estimated that alcohol-related harm costs society over £1 billion in Wales each year [3]. The World Health Organization notes that there is no safe level or pattern of alcohol use; nevertheless, guidelines in the UK highlight that alcohol consumption should not exceed 14 units (one unit is 10 mL or 8 g of pure alcohol) a week, and that consumption should be spread over three or more days [4].

The risk of alcohol-related harm increases as alcohol consumption increases [1]. The relationship between alcohol consumption and alcohol-related harms is often illustrated using a J-shaped curve, with moderate consumption appearing least harmful. However, this model has been questioned because it overlooks confounders, including the fact that many abstainers are former heavy drinkers who stopped drinking due to alcohol-related harms [5]. The relationship between alcohol consumption and socioeconomic status is complex and does not necessarily follow a linear trend. Nevertheless, the existing literature consistently implies that more deprived areas experience higher levels of alcohol-related harms despite having similar levels of alcohol consumption to less deprived areas [6,7]. In a literature review conducted by Jones et al. [8], it was found that individuals of lower socioeconomic status were nearly twice as likely to experience an alcohol-related death or hospitalisation. Health inequalities are well established in the healthcare literature, where populations of lower socioeconomic status are more likely to die or suffer from illnesses and diseases. This also extends to alcohol-related harms. This phenomenon is known as the alcohol-harm paradox, a worldwide recognised phenomenon which has been researched across the globe including in the United Kingdom [7,9], Finland [10], Norway [11] and Latin America [12].

The alcohol-harm paradox is an example of Simpson’s paradox, a statistical paradox where a trend observed within several groups of the data disappears or reverses when the groups are combined. It is known that alcohol-related harms increase with alcohol consumption; however, in certain populations this trend is reversed. This reversal is associated with confounders.

Numerous confounders have been researched or proposed to explain the alcohol-harm paradox as the increased level of harm amongst lower socioeconomic populations has generally not been able to be attributed to increased alcohol consumption [13]. Table 1 summarises the factors that have been considered, some of which may help to explain why less deprived areas consume more units of alcohol annually, whereas others may explain why more deprived areas experience more alcohol-related harms.

Health behaviours including smoking, diet, physical activity and BMI are frequently reported as potential factors to explain the alcohol-harm paradox. Research has found health-risk behaviours such as smoking, being overweight, having a poor diet and low exercise level to all be associated with increased risk of alcohol-related harms [9,14]. However, findings are mixed as some studies suggest that differences in alcohol-related harms by deprivation cannot be accounted for by differences in BMI and smoking [7]. Boyd et al. [13] conducted a literature review and identified a range of proposed explanations, highlighting risk behaviours as being central, but insufficient, to explain the paradox, due to its multifactorial nature. Furthermore, alcohol use often co-occurs with other substance use [15].

**Table 1:** Summary of explanations for the alcohol-harm paradox explored or proposed in the literature.

Category	Factor	Citations
Access to support services		[8,16,17]
Age		[18,19]
Availability of venues	Number/Density	[20,21,22]
	Trading hours	[20,22]
	Distance	[22,23,24]
Consumption behaviours	Type	[9,14,25,26]
	Quantity	[8,27,28,29]
Deprivation domains	Income	[7,27,30,31,32,33,34]
	Education	[35,36,37,38,39,40]
Other health behaviours		[8,9,14,27,34]
Reverse causation		[7]
Rural/Urban		[41,42,43]
Under-reporting		[8,44,45]

The type of alcoholic beverage consumed has been suggested as a confounder in the alcohol-harm paradox [13]. Beer and cider consumption have been shown to be more prevalent amongst deprived drinkers and associated with binge drinking, whereas those that chose wine tended to be from less deprived areas and were more likely to exceed weekly unit intake guidelines [8,9]. Another hypothesised confounder is the quantity of alcohol consumed per drinking session. Binge drinking has consistently been found to be more prevalent in deprived individuals [9,27,28], although total alcohol consumption does not differ from their affluent counterparts. It has been suggested that deprived individuals are more likely to under-report their alcohol consumption; however, evidence remains limited [8,9].

Deprivation has been measured by different domains such as income and education to explore the best predictors of alcohol consumption and alcohol-related harms. Higher levels of binge drinking have been shown to be associated with lower educational attainment [8], with education being a good predictor of consumption frequency and quantity [35].

Some research has found that more deprived areas have a higher alcohol outlet density; however, the relationships between alcohol outlet density, alcohol consumption, and alcohol-related harms remain unclear [8]. Mehranbod et al. [46] propose that individuals travel to alcohol outlets beyond their local area to address these conflicting findings. Sherk et al. [20] conducted a literature review where 90.91% of studies concluded that restricting the physical availability of take-away alcohol would reduce individual alcohol consumption.

An alternative measure of access to alcohol is proximity. Halonen et al. [47] found that the likelihoods of both heavy alcohol use and extreme drinking occasions were higher amongst individuals that lived less than one kilometre from a bar. Similarly, Hay et al. [48] found that travel distances to alcohol venues were 50% greater in the least deprived areas in comparison to the most deprived areas; however, this relationship was different for urban and rural areas. A key limitation of research on the geography of alcohol outlets is the tendency to aggregate venues into a single category, disregarding differences between on- and off-licensed outlets and the variation within them [20,22].

Motivation for consumption, and the type and quantity of alcohol consumed, may differ depending on the venue in which alcohol is purchased. Consuming alcohol at a pub, bar or restaurant suggests a social context, whereas purchasing alcohol from a convenience store might be indicative of consumption at home and alone [49,50]. Reasons given for drinking at home include convenience, cost, safety, social occasion, and stress relief or reward [50].

In summary, the related work suggests a range of possible factors in the alcohol-harm paradox. However, these factors have typically been analysed separately. This research considers some of the conflicting findings in the related work, and addresses the gap in the literature by researching the role of numerous influences in one region.

This research aims to use open-access data to explore the most prevalent factors in the literature to hypothesise the factors associated with the alcohol-harm paradox in Gwent, a region in South-East Wales. This is a region where the more deprived areas experience higher rates of alcohol-related harms despite having lower annual alcohol consumption. Furthermore, Gwent constitutes a socioeconomically diverse region, encompassing both some of the most and least deprived areas in Wales, providing a representation of deprivation patterns across Wales. Gwent’s socioeconomic diversity creates a wide socioeconomic gradient which is necessary to explore whether deprivation is associated with disproportionate alcohol-related harm. Additionally, studies of alcohol consumption and its consequences are often researched using a regional scope [51]. Previous studies have often only considered some of the factors proposed to explain the alcohol-harm paradox, and considered only one measure of alcohol consumption as they usually used a new study sample. This research explores a range of factors, including demographics, health behaviours, and density and proximity of alcohol outlets, in association with both average annual alcohol consumption and binge drinking prevalence. Binge drinking is defined as consuming more than eight units for men, or more than six units for women, in a single drinking session. This research aims to answer the following research questions:

1. Is there evidence of the alcohol-harm paradox in Gwent?
2. What factors are associated with the alcohol-harm paradox in Gwent?
3. How can the findings be applied beyond Gwent?

The remainder of the paper is structured as follows: Section 2 outlines the data sources and methodology used. Section 3 presents the findings of the exploratory analysis such as the prevalence of health behaviours. Section 4 discusses the implications of the findings. Finally, Section 5 presents the conclusions of the paper and offers directions for future work and interventions.

## 2. Materials and Methods

This research explores the alcohol-harm paradox in Gwent, a region in South-East Wales, and explores the influence of factors proposed to explain the paradox. The exploratory analysis of cross-sectional secondary data sources is used to identify potential associations and formulate hypotheses. Analyses are primarily descriptive and exploratory. This research is reported in accordance with the STROBE guidelines for cross-sectional observational studies, shown in Supplementary Section S3.

**Figure 1:**
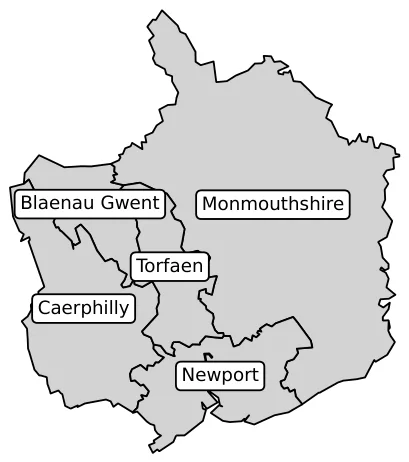
Map of Gwent and its five local authorities.

Gwent contains five local authorities: Blaenau Gwent, Caerphilly, Monmouthshire, Newport, and Torfaen, as shown in Figure 1. The region’s population is estimated to be 601,686 [52]. Gwent has a diverse socioeconomic profile, constituting of some of the most and least deprived areas in Wales. It includes densely populated urban areas such as Newport and industrial history in areas such as Blaenau Gwent, as well as more rural areas, namely, Monmouthshire.

A range of open-access data sources were used to explore the alcohol-harm paradox in Gwent, including national population surveys, healthcare data, and geospatial data. All analysis and code is archived at [53], and all data is archived at [54].

### 2.1. National Survey for Wales

The National Survey for Wales (NSW) is the source of the majority of the data used in this research. The survey is designed to be representative of adults 16 years and older living in private households in Wales. The sample is drawn from the Royal Mail Small Users Postcode Address File, and ensures that a minimum effective sample size is achieved in each local authority. Once a household has been selected, an adult aged 16+ is randomly selected to participate in the survey. The yearly survey involves around 12,000 participants.

The survey covers a range of topics including housing, NHS and social care, and the local environment. The main topic of interest for this research was population health, specifically, adult lifestyles which covers smoking, alcohol consumption, diet, exercise and BMI [8,9,14,27,34]. Alcohol consumption is categorised by average weekly alcohol consumption, defined as none, moderate (up to 14 units), hazardous (over 14 units, and up to 50 for males and 35 for females) or harmful (over 50 units for males and 35 for females). The NSW was also used to explore alcohol consumption across sub-groups such as age and sex because demographics can shape both consumption behaviours and susceptibility to harm [18,19]. The most recent data from the NSW is from 2022/23, where data collection was conducted with 11,140 participants, of which 2075 were from Gwent, from April 2022 to March 2023. Only 868 respondents from the Gwent sub-sample were able to be categorised into the aforementioned consumption groups. The NSW data was weighted using the health lifestyle sub-sample weight.

Up until 2019/20, the NSW included a question regarding maximum alcohol consumption on the heaviest drinking day in the past seven days. This question was altered following the introduction of telephone surveys [55]. Consequently, the latest binge drinking data is from 2019/20.

Annual consumption and binge drinking data were not available at the LSOA level. The most granular data that could be obtained was stratified by overall deprivation quintile within each local authority. As Gwent comprises 5 local authorities, there were 25 data points in total (5 deprivation quintiles per local authority). Each LSOA within the same local authority and deprivation quintile was assigned the corresponding annual alcohol consumption and binge drinking prevalence.

### 2.2. Welsh Index of Multiple Deprivation (WIMD)

The WIMD is the official measure of relative deprivation in Wales, produced by the Welsh Government. Every LSOA in Wales is ranked based on its deprivation from 1 (most deprived) to 1917 (least deprived) [56]. Each LSOA has a population of approximately 1600. The Welsh Government defines deprivation as “the lack of access to opportunities and resources which we might expect in our society”, where this could be material goods, or the ability to participate in every day social life [57]. The WIMD does not provide a measure of deprivation or affluence; rather, it provides a ranking, allowing comparison of deprivation of one LSOA with another. The WIMD is made up of eight domains: income, employment, health, education, access to services, housing, community safety, and physical environment, each assigned a weight (see Supplementary Section S2) based on the importance of the domain as a component of deprivation, and the quality of the indicators for the domain. WIMD analyses were grouped by deprivation quintile to enable comparison across socioeconomic divisions.

### 2.3. Alcohol-Related Harms

Alcohol-related harms are typically measured in two ways in the context of the alcohol-harm paradox—deaths and hospital admissions. The Office for National Statistics provides yearly data on the number of alcohol-specific deaths [58,59]. Mortality data for all deaths recorded in 2023 were used. Alcohol-specific deaths only include the health conditions where the death is a direct consequence of alcohol. Digital Health and Care Wales provides yearly data on alcohol hospital admission rates. Admissions can be categorised as alcohol-specific (admission for alcohol-specific conditions, either in the primary diagnosis or in secondary diagnoses), alcohol-attributable narrow measure (primary diagnosis was an alcohol-attributable condition, or one of the secondary diagnoses was an external cause with an alcohol-attributable fraction), or alcohol-attributable broad measure (hospital episodes with any mention of an alcohol-attributable diagnosis in any diagnostic position). The broad measure was used in this analysis to assess the broader burden of alcohol consumption, and to compensate for under-reporting. Admissions data for 2022/23 was used. Deaths and hospital admissions were expressed as rates per 100,000 of the population to allow comparisons across local authorities of differing population size.

### 2.4. Ordnance Survey

Alcohol-related venues in and around Gwent were identified through the Points of Interest Ordnance Survey dataset, accessed through DigiMaps. The Points of Interest dataset provides a comprehensive record of the location of all businesses, education and leisure services in Great Britain [60]. Venues are classified using a three-level classification scheme. The groups, categories and classes included in this research are provided in Supplementary Section S1.

To calculate outlet density, only venues in the five local authorities in Gwent were included in the analysis. For distance measures, venues outside Gwent were also included to account for travelling to another region. Distance was computed by LSOA, and was calculated as the distance from the centroid of each LSOA to the nearest alcohol-related venue. Average distance to the nearest *k* venues was also calculated to provide a more robust measure. The nearest 10 venues were considered in most cases, unless there was only a small number of a type of venue, then only the nearest five venues were considered. Both on- (pubs, bars and inns, nightclubs, social clubs, restaurants, and hotels) and off-licenced (alcoholic drinks retailers, convenience stores, supermarkets, and drinks manufacturers) premises were explored to address limitations in previous research [20,22]. On-licence allows the sale of alcohol for consumption on the premises, whereas off-licence allows the sale of alcohol for consumption off the premises. To visualise outlet proximity, distance to venues was calculated and subsequently divided into quintiles based on the distribution of observed values. The Pearson correlation coefficient was calculated to test the relationship between the number of, or the distance to, alcohol-related venues and alcohol consumption, binge drinking, and alcohol-related harms. A significance level of *p* < 0.05 was used for all statistical analyses.

### 2.5. Multinomial Logistic Regression

Multinomial logistic regression modelling was used to assess the relationship between alcohol-harm paradox variables and deprivation, and to explore deprivation-related differences in health behaviours. Multinomial logistic regression is a generalised logistic regression method for multiclass problems. Regression methods have commonly been used to explore the relationship between deprivation, alcohol harms, and factors hypothesised to be associated with the alcohol-harm paradox [7,9,11,18,29]. Multinomial logistic regression models were used to estimate the odds of having an unhealthy behaviour, drinking specific types of alcohol, and binge drinking a specific type of alcohol in deprived compared with non-deprived populations, with each factor modelled separately. Using NSW data, quintiles 1 and 2 were classed as deprived, while quintiles 3, 4, and 5 were classed as non-deprived, consistent with previous research [7]. Separate models were constructed for each unhealthy behaviour, each type of alcoholic drink consumed, and for the quantity of each type of alcoholic drink consumed. For health behaviours, the baseline category was defined as the ‘healthy’ variant of each behaviour. For alcohol consumption, the baseline for the type of beverage was non-consumption of each beverage, while the baseline for consumption quantity was defined as ‘not binge drinking’. The multinomial logistic regression equation is as follows:

(1) $$\ln\frac{P(Y=k)}{P(Y=0)}=\beta_{k0}+\beta_{k1}x$$

The independent variable, *x*, is deprivation, and the dependent variable *Y* is a categorical variable indicating an unhealthy behaviour, type or quantity of alcohol consumed, where 0 is the baseline category. The parameter *k* represents the number of possible outcomes, and we have k−1 regression equations. For example, for smoking, we have three classes: smoker, ex-smoker, and never smoked. The reference category, Y=0, would be never smoked, and k=1,2 represent the classes ex-smoker and smoker.

## 3. Findings

This section presents the findings of the exploratory analysis using the datasets outlined above. The analysis aimed to explore the influence of potential factors in the alcohol-harm paradox, and address the research questions outlined in Section 1.

### 3.1. Is There Evidence of the Alcohol-Harm Paradox in Gwent?

**Figure 2:**
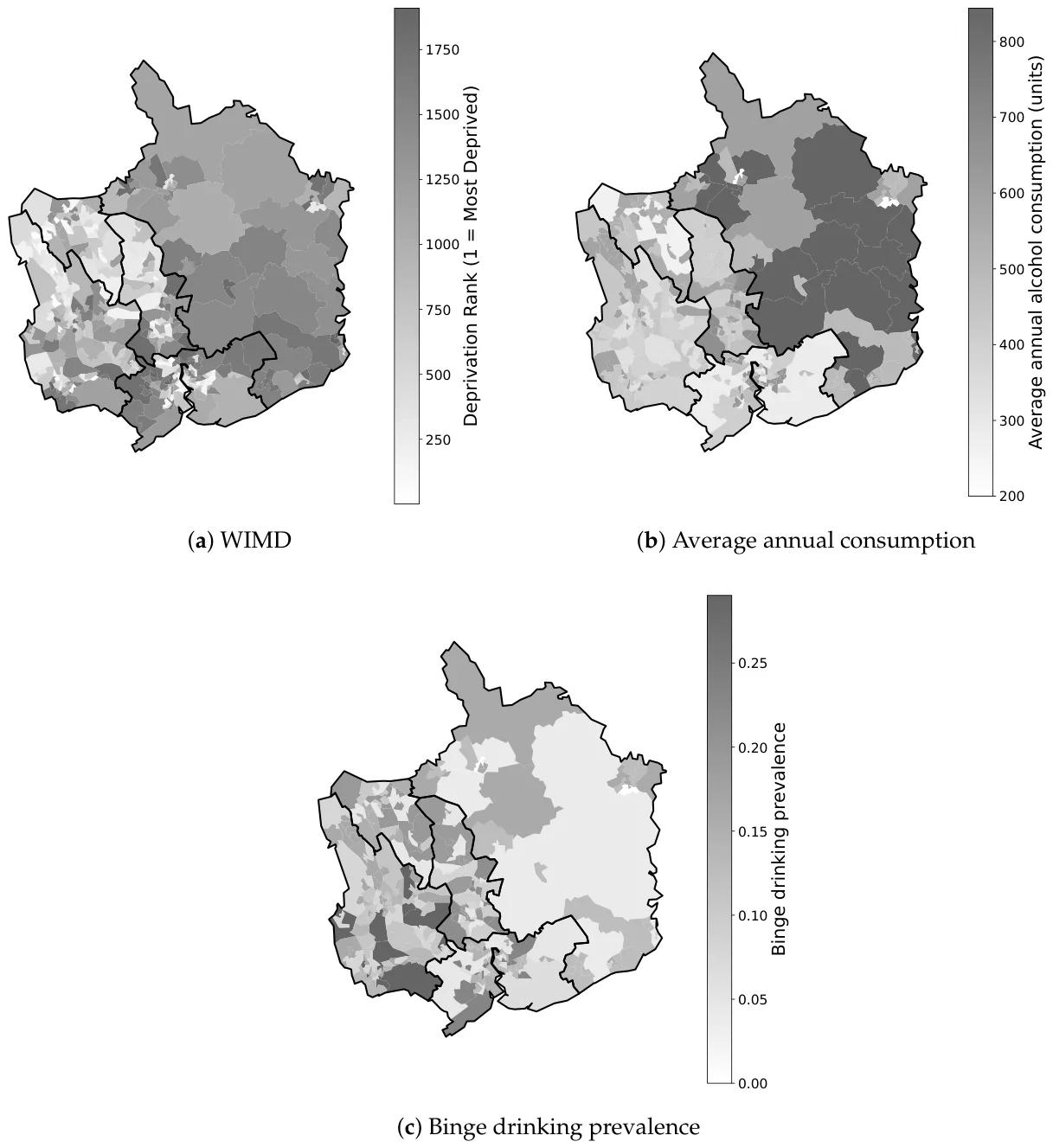
Choropleth maps of WIMD, annual alcohol consumption, and binge drinking prevalence by LSOA.

Figure 2 presents choropleth maps of the WIMD, average annual alcohol consumption, and binge drinking prevalence by LSOA. WIMD ranking was available by LSOA, whereas alcohol consumption and binge drinking data were available stratified by local authority and deprivation quintile. The maps indicate geographic variation in alcohol consumption and binge drinking prevalence across deprivation levels.

Firstly, the existence of the alcohol-harm paradox in Gwent was verified. Table 2 reveals the annual alcohol consumption by the least (Monmouthshire), moderately (Caerphilly and Torfaen), and most (Blaenau Gwent and Newport) deprived local authorities in Gwent. Grouping local authorities into broader deprivation categories allowed for clearer exploration of the socioeconomic gradient while increasing interpretability. Population statistics indicate that annual consumption is higher in the least deprived area. Overall in Wales, the most deprived areas consume more alcohol annually; however, the difference is subtler, with annual consumption of 540 and 493 units in the least and most deprived areas, respectively, further reinforcing the diverse consumption across Gwent.

Secondly, the association between deprivation and the rate of alcohol-related harms was explored to investigate whether the main theory of the alcohol-harm paradox exists in Gwent, where greater levels of harms are experienced in more deprived areas. Table 2 reveals that the most deprived areas experience higher rates of both alcohol-specific deaths and alcohol-attributable hospital admissions. Interestingly, there is not much difference in the alcohol-attributable hospital admission rates of the moderately and most deprived areas. The prevalence of alcohol-related harms was compared to alcohol consumption to verify whether consumption itself drives alcohol-related harms, or whether deprivation modifies the relationship between consumption and harms. Table 2 shows that the area with higher alcohol consumption experiences a lower alcohol-specific death and alcohol-attributable hospital admission rate. The role of binge drinking was also explored to provide an alternative measure of alcohol consumption. Table 2 reveals that binge drinking prevalence is highest in moderately deprived areas.

### 3.2. Demographics

Having observed preliminary evidence of the alcohol-harm paradox in Gwent, the factors hypothesised to be causing the paradox were explored. The first hypothesis explored was demographics, which included measures of socioeconomic status, age, sex, and a measure of urban or rural.

#### 3.2.1. Alternative Domains of WIMD

**Figure 3:**
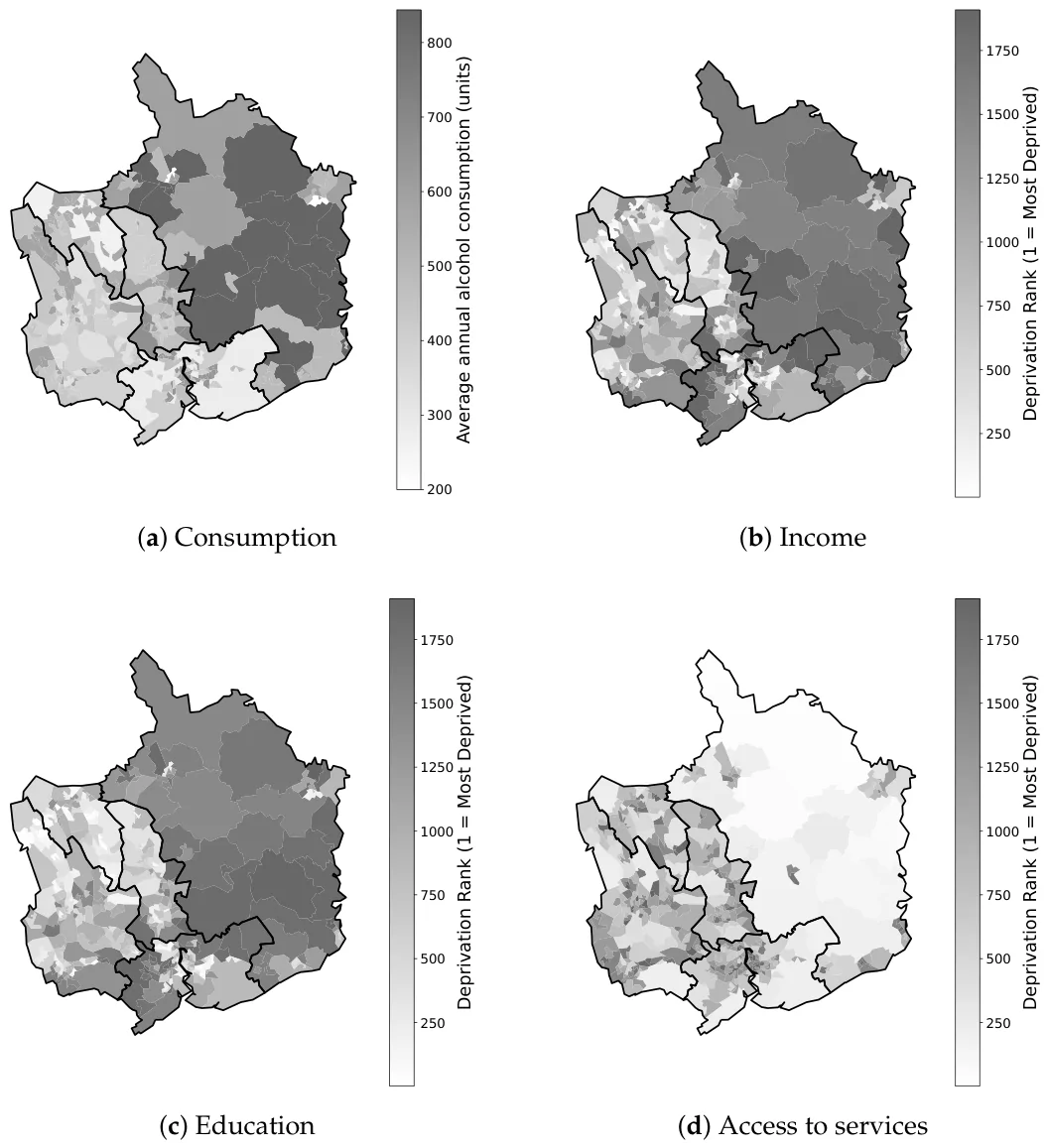
Domains of WIMD across LSOAs in Gwent.

Deprivation can be measured by different domains, and three of these are presented in Figure 3 along with annual alcohol consumption. Visually, income deprivation seems to have a negative association with average annual alcohol consumption, where areas with greater alcohol consumption experience lower income deprivation. Education, employment and health also appear to follow the same trend as income (see Section S2). Interestingly, ‘access to services’ is the only domain that looks to follow the same trend as annual alcohol consumption.

#### 3.2.2. Age

Age distribution can be measured by different metrics, such as mean and median age, or proportions in important age brackets. This research considered the mean age and proportion of the population of each local authority aged 65+. Monmouthshire has the highest mean age as well as the largest proportion of individuals aged 65+, whereas Newport has both the lowest mean age and smallest proportion of individuals aged 65+ (see Supplementary Section S4). Blaenau Gwent, Caerphilly and Torfaen have relatively similar age compositions.

Average annual alcohol consumption and binge drinking prevalence was explored by age group (see Section S4 for detail). Annual alcohol consumption increases with age up until age 45–64 and plateaus thereafter. Binge drinking prevalence shows the opposite trend to annual alcohol consumption, with binge drinking prevalence decreasing as age increases.

#### 3.2.3. Urban and Rural

Urban–rural classification was considered in terms of population density, measured as the number of residents per square kilometre. The greatest difference in population density was observed between Monmouthshire and Newport (see Supplementary Section S5 for more detail). The association between population density and annual alcohol consumption, and between population density and binge drinking prevalence, was explored at the local authority level by using Pearson’s correlation coefficient. A negative correlation was observed between population density and annual alcohol consumption (ρ = −0.871, *p*-value = 0.054), while a positive correlation was observed between population density and binge drinking prevalence (ρ = 0.798, *p*-value = 0.105).

#### 3.2.4. Sex

Differences in average annual alcohol consumption and binge drinking prevalence were observed between males and females, with average annual alcohol consumption of 679.3 and 279.4 units, and binge drinking prevalence of 17.6% and 10.8%, respectively.

The distribution of alcohol consumption, defined by weekly units, was explored by sex. A greater proportion of females abstain or consume alcohol under the recommended guidelines in comparison to males, whereas a greater proportion of males consume alcohol at hazardous or harmful levels in comparison to females (see Supplementary Section S6). There is no significant difference in the proportion of males and females in the local authorities in Gwent, with 48.5% being male and 51.5% being female. There is also no significant difference in the distribution of males and females by deprivation quintile in Gwent.

### 3.3. Health Behaviours and the Type and Quantity of Alcohol Consumed

In the literature, health behaviours are commonly reported as potential influential factors [8,9,14,34], where health behaviours refer to actions taken to influence an individual’s health, typically including alcohol consumption, smoking, diet, BMI and physical activity.

Table 3, ordered from least to most deprived, displays the health behaviour distribution of each local authority in Gwent. Variability in health behaviours was observed across the local authorities, with unhealthy behaviours predominantly concentrated in Blaenau Gwent. Blaenau Gwent has the highest proportion of the population consuming no fruit or vegetables in the previous week, not meeting weekly exercise guidelines, and that are overweight or obese. Torfaen has the highest smoking prevalence.

The health behaviours in Table 3 can be summarised into one measure, which is the number of healthy behaviours, defined as being a non- or ex-smoker, eating 5+ portions of fruit or vegetables, undertaking 150+ minutes of weekly exercise, or having a healthy BMI. Table 3 reveals the proportion of each local authority with each number of healthy behaviours. Blaenau Gwent is the local authority with the smallest proportion of its population having five healthy behaviours, in addition to being the local authority with the largest proportion of its population having no healthy behaviours. Conversely, Monmouthshire, the local authority with the lowest rate of alcohol-related harms, has the largest proportion of its population with four or five healthy behaviours.

Table 4 provides the results of the separate univariate multinomial logistic regression models for each health behaviour. Smoking, being an ex-smoker, being obese, poor diet and drinking wine each showed statistically significant odds ratios, indicating that the odds of exhibiting these unhealthy behaviours, relative to the corresponding healthy behaviour, differed significantly between deprived and non-deprived populations. These models were not adjusted for potential confounders.

Another hypothesis behind the alcohol-harm paradox is the effect of the type of alcohol consumed [8,9,14,25,26]. In this research, alcohol was categorised as beer, wine or spirits. Deprivation was significantly associated with wine consumption, the only statistically significant odds ratio observed for the type of alcohol consumed.

Thirdly, the quantity of different types of alcohol consumed, categorised as binge or not binge drinking, was considered. No statistically significant associations were observed between deprivation and the quantity of each type of alcohol consumed.

### 3.4. Geographical Mapping of Alcohol-Related Venues

The final hypothesis explored was the geography of alcohol-related venues. The geographical landscape of alcohol-related venues was explored in two ways: outlet density and proximity to outlets. These measures were explored by considering a range of on- and off-licenced premises.

Newport has the greatest number of alcohol-related venues, followed by Caerphilly, then Monmouthshire (Table 5, ordered from least to most deprived). Blaenau Gwent and Torfaen both have less than half the number of alcohol-related venues compared to Newport, Caerphilly and Monmouthshire. Convenience stores account for the highest proportion of venues in Newport, Blaenau Gwent, Caerphilly and Torfaen. However, pubs, bars and inns account for the greatest proportion in Monmouthshire, accounting for 31.3% of the venues in the local authority.

#### 3.4.1. Number of Venues per Local Authority and LSOA

Firstly, alcohol outlets were explored in terms of outlet density, where the number of venues was considered per LSOA and km^2^ in each local authority in Gwent. Monmouthshire, the local authority with the highest annual alcohol consumption and lowest binge drinking prevalence, has the highest count of venues per LSOA whilst having the smallest population density. Torfaen, the local authority with the highest binge drinking prevalence and relatively high alcohol consumption, has the smallest count of venues per LSOA. Monmouthshire and Torfaen have the smallest count of venues per km^2^. For more detail, see Supplementary Section S7.

Statistically significant Pearson correlation coefficients were found between alcohol-specific death rates and the number of pubs and hotels at the local authority level (ρ=−0.94, *p*-value = 0.02, ρ=−0.93, *p*-value = 0.02, respectively). No statistically significant associations were found between outlet density and alcohol-attributable hospital admission rates.

The number of alcohol-related venues per LSOA was also explored by deprivation quintile, and it was revealed that quintiles 1 and 4 both have the highest outlet density per LSOA (see Supplementary Section S7). Convenience stores comprise the largest proportion of venues in quintile 1, representing 47.4% of all venues. The largest proportion of venues in quintile 4 consists of pubs, bars and inns, representing 33.6% of the venues.

#### 3.4.2. *k* Nearest Alcohol-Related Venues

The distance to the *k*-nearest alcohol-related venues was calculated to provide a robust measure of proximity to venues. Results are presented for the four types of venues hypothesised to be most influential, with more detail provided in Section S8. Newport, the local authority with the lowest average annual alcohol consumption, is closest to all types of alcohol-related venues, except for pubs. Conversely, Monmouthshire, the local authority with the highest average annual alcohol consumption, is furthest away from all types of alcohol-related venues, except for pubs. Blaenau Gwent and Torfaen, the local authorities with the highest alcohol-specific death rates, are the local authorities closest and furthest away from pubs, respectively. Pearson correlation coefficients were calculated to test the association between distance to the nearest *k* venues and alcohol consumption at the local authority level. Statistically significant positive associations were found between distance to the nearest nightclubs, social clubs and convenience stores (ρ=−0.93, *p*-value = 0.02, ρ=−0.99, *p*-value = 0.00, ρ=−0.99, *p*-value = 0.00, respectively). The association between distance to the nearest *k* venues and alcohol-related harms was also tested, and a statistically significant association was found between distance to the nearest 10 convenience stores and alcohol-attributable hospital admissions (ρ=−0.89, *p*-value = 0.04). No statistically significant associations were found between distance and alcohol-specific deaths.

Variation in outlet proximity by deprivation quintile was observed. The most deprived LSOAs are closer to alcohol-related venues, with social clubs showing the clearest gradient (see Section S8). Pub proximity varied little across deprivation quintiles.

**Figure 4:**
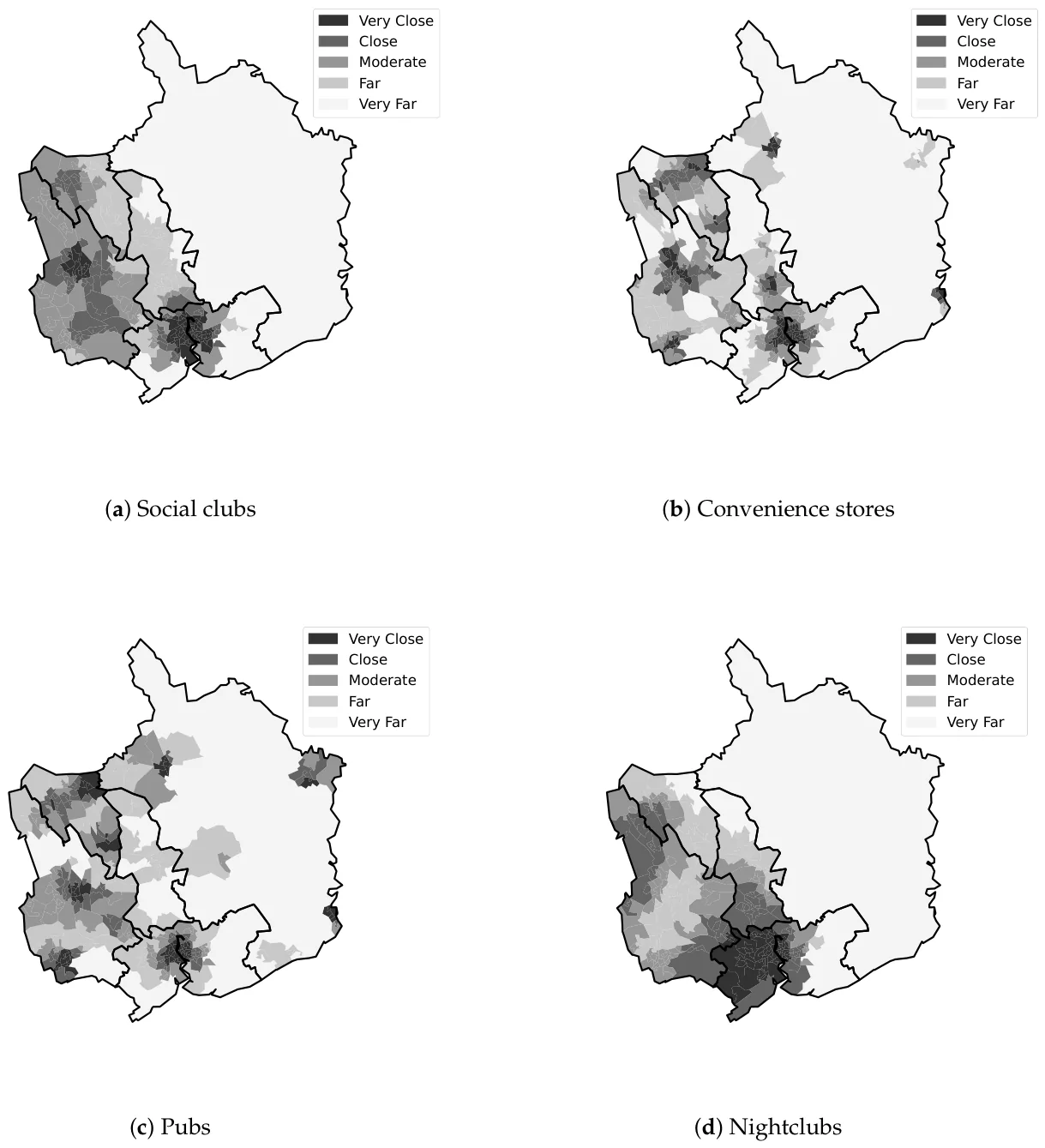
Choropleth maps of distance to the nearest 5 or 10 venues.

When visually exploring average distance to the *k* nearest social clubs, portrayed in Figure 4, there are two main hotspots—one in Caerphilly, and one in Newport. A cluster of smaller distances is also seen in Blaenau Gwent. These are the local authorities with the lowest average annual alcohol consumption, and highest alcohol-attributable hospital admission rates.

The map portraying the distance to the five nearest nightclubs looks similar to the map for social clubs; however, there are only two hotspots of nightclubs—one in Newport and the other mainly in Caerphilly. The map of distances of the nearest 10 convenience stores shows multiple hotspots of convenience stores across Gwent, similar to the map for pubs. Associations between average distances to the nearest *k* venues and binge drinking prevalence were explored; however, no statistically significant correlation coefficients were found.

## 4. Discussion

This research presents a data-driven exploratory analysis of the alcohol-harm paradox in Gwent, a region in South-East Wales. As this study is cross-sectional, causal relationships between alcohol consumption and alcohol-specific deaths and alcohol-attributable hospital admissions cannot be inferred; therefore, the findings represent associations at a single point in time and may be influenced by unmeasured confounders. The research presents an exploratory analysis in one region; therefore, the findings should be interpreted as hypotheses requiring further testing elsewhere.

### 4.1. Is There Evidence of the Alcohol-Harm Paradox in Gwent?

Preliminary analyses suggested a socioeconomic gradient in alcohol consumption behaviours in Gwent, where more deprived areas reported lower average annual alcohol consumption but higher binge drinking prevalence, while less deprived areas showed the opposite pattern, suggesting that higher levels of deprivation are not associated with greater alcohol exposure. Higher rates of both alcohol-specific deaths and alcohol-attributable hospital admissions in more deprived areas support the presence of the alcohol-harm paradox in Gwent. The broad definition of alcohol-attributable hospital admissions used may over-attribute in some cases; however, it provides a more comprehensive estimate of the true burden of alcohol consumption, and overcomes the fundamental under-reporting in alcohol data. Although the literature notes that the risk of alcohol-related harm increases as consumption increases [1], an inverse association between alcohol consumption and alcohol-related harms was observed in Gwent, likely reflecting the influence of deprivation and other socioeconomic, demographic and behavioural confounders as opposed to a protective effect of alcohol consumption.

Binge drinking portrayed the opposite socioeconomic pattern to alcohol consumption, where prevalence was lowest in the least deprived areas of Gwent. This suggests that drinking patterns, rather than consumption volume alone, may contribute to alcohol-related health inequalities, consistent with the previous literature [8,25,27]. Consequently, policies focusing on annual alcohol consumption may fail to target problematic high-risk drinking. However, binge drinking estimates were based on 2019/20 data. This is a key limitation of this research as consumption behaviours are believed to have changed following the COVID-19 pandemic [61,62]. Nevertheless, the findings highlight the importance of collecting both annual alcohol consumption and binge drinking data in future. This exploratory analysis should be replicated when more current data becomes available.

### 4.2. Demographics

Ocular comparison suggested that income and education deprivation were negatively associated with alcohol consumption in Gwent, potentially reflecting greater disposable income among less deprived individuals to spend on alcohol. Educational attainment may influence the ability to mitigate alcohol-related harms through access to health-related resources, suggesting that interventions could target populations with lower educational qualifications [35,39]. Nevertheless, income reporting can be susceptible to non-response bias due to its sensitive nature [63], and WIMD domains are strongly correlated [64], limiting interpretation of individual domain effects. Contrary to expectations, visual inspection suggested that ‘access to services’ deprivation might be positively associated with alcohol consumption which is likely influenced by the rurality of Monmouthshire.

Age was also explored as a possible contributing factor. Monmouthshire had both the highest average age and highest average annual alcohol consumption, suggesting a possible association between age and alcohol consumption in Gwent. However, as alcohol consumption contributes to the premature onset of health complications [65], age alone is unlikely to fully explain the alcohol-harm paradox. Middle-aged adults reported higher annual alcohol consumption whereas binge drinking was more prevalent amongst younger populations. Nevertheless, the age-related consumption findings are likely sensitive to individual observations due to the small sample size for the 16–24 age group in the NSW. Furthermore, NSW’s 16–24 age band combines individuals above and below the legal drinking age of 18 in Wales; therefore, the drinking behaviours of 16- and 17-year-olds should be considered separately.

No analyses using typical urban–rural classification labels were included as these labels were considered to be a reductionist approach, especially with only five local authorities, and only one of these typically being considered as rural, namely, Monmouthshire.

While males exhibited higher levels of hazardous and harmful alcohol consumption, the gender composition of Gwent’s local authorities was relatively even, thereby mitigating the influence of sex on the alcohol-harm paradox in Gwent. The survey results do not exclude pregnant women; therefore, we cannot determine whether differences in the ‘None’ category are attributable to women abstaining during pregnancy.

### 4.3. Health Behaviours and the Type and Quantity of Alcohol Consumed

Unhealthy behaviours appeared to be concentrated within local authorities exhibiting the highest rates of alcohol-specific deaths and alcohol-attributable hospital admissions, suggesting that alcohol-related harms may be embedded within broader patterns of unhealthy behaviours in Gwent. Univariate multinomial logistic regression models suggested that deprivation was associated with increased odds of each unhealthy behaviour, except for exercise. However, these models should be interpreted cautiously as they were univariate and unadjusted, and, therefore, did not account for potential confounding variables such as age, sex or education, and multicollinearity between unhealthy behaviours was not explored due to limitations in the available data. Nevertheless, the findings are broadly consistent with previous hypotheses that deprived individuals’ elevated levels of alcohol-related harms may be associated with the accumulation of other health risk behaviours [9,13]. Bellis et al. [9] suggest that public health messaging could focus on how a combination of unhealthy behaviours may exacerbate alcohol-related harms. Alcohol-related harms may be more effectively addressed through integrated public health interventions targeting multiple health-risk behaviours simultaneously.

For alcohol type, the only statistically significant odds ratio observed was for wine consumption, with more deprived individuals having lower odds of consuming wine compared with less deprived individuals, consistent with previous research [8]. Wine consumption has historically been associated with social norms, consumption with meals, and moderate consumption behaviours [66,67,68]. Differences in alcohol type may therefore contribute to socioeconomic variations in alcohol-related harms in Gwent, rather than any protective effect of wine, although this analysis could not establish whether this association was independent of confounders.

### 4.4. Geographical Mapping of Alcohol-Related Venues

This research extends previous work by exploring both alcohol outlet density and proximity. Previous studies have typically focused on one measure of alcohol availability [20,69]. Furthermore, both on- and off-licenced premises were included, addressing limitations in the range of venue types considered in previous research [20,22].

Differences in alcohol venue composition were observed. Monmouthshire, which has the lowest rates of both alcohol-specific deaths and alcohol-attributable hospital admissions, contained a greater proportion of pubs, bars and inns, which may indicate more regular social drinking, as suggested in the literature [49]. In contrast, convenience stores comprised the majority of venues in all other local authorities, suggesting that venue type, rather than density alone, may contribute to the paradox in Gwent. Hay et al. [48] note that increasing the prevalence of licenced restaurants may help to shift focus towards food rather than alcohol consumption alone. Additionally, differences in venue composition suggest that licensing and urban planning approaches may play a role in reducing alcohol-related harms.

Although Monmouthshire had the highest count of venues per LSOA and the highest annual alcohol consumption, associations between outlet density and alcohol consumption were inconsistent, aligning with previously reported mixed evidence regarding the influence of outlet density [8]. Monmouthshire has the smallest population density, resulting in a larger number of venues per person. As LSOAs have roughly the same population size, comparing the number of venues per LSOA across local authorities is approximately equivalent to comparing the number of venues per person. Nevertheless, Monmouthshire had fewer venues per km^2^, suggesting that the higher number of venues per LSOA may reflect its rurality rather than greater alcohol availability. No statistically significant correlations with alcohol-attributable hospital admission rates were found, contrary to other studies [69]. However, with only five local authorities, correlation coefficients are likely to be unstable and sensitive to individual observations.

Findings relating to outlet density and deprivation quintile suggest a complex relationship between deprivation and outlet density, consistent with the previous literature [8]. Convenience stores were particularly prevalent in quintile 1 LSOAs, suggesting that off-licence availability may contribute to alcohol-related harms in Gwent, potentially through greater affordability. Nevertheless, convenience stores might not be a direct proxy for alcohol consumption as they can be used to purchase other groceries. In contrast, pubs, bars and inns were prevalent in quintile 4 LSOAs, further supporting suggestions that socially oriented drinking environments may provide a more social and less harmful drinking context [49].

Findings relating to local authority proximity to alcohol-related venues were mixed. Newport’s close proximity to alcohol-related venues may only reflect its urban character, higher population density, city status and relatively younger population profile. Monmouthshire was furthest away from all venues, except for pubs, whilst also having the highest average annual alcohol consumption. Blaenau Gwent and Torfaen, the local authorities with the highest alcohol-specific death rates, were the closest and furthest away from pubs, respectively, further obscuring any proximity associations. Statistically significant positive correlations between proximity to nightclubs, social clubs and convenience stores, and alcohol consumption, suggest that proximity to alcohol-related venues is a paradox within itself. A positive correlation between proximity to convenience stores and alcohol-attributable hospital admissions may suggest that spatial access to specific venue types may contribute to alcohol-related harms in Gwent, although, as noted previously, convenience stores might not be a direct proxy for alcohol consumption. While causal relationships cannot be inferred, the findings may have implications for local licensing objectives. Nevertheless, these findings should be interpreted cautiously, as analyses were unadjusted to drinking motivations, social context, and drinking setting, and proximity to a venue does not necessarily imply use of that venue. Consequently, spatial associations identified should be interpreted as exploratory. Future work could conduct formal spatial modelling to address autocorrelations.

Local authorities in Gwent with higher alcohol-attributable hospital admissions were closer to social clubs, venues traditionally associated with cheaper drinks, industrial communities and a place for socialising [70]. Gwent has historically been recognised as an industrial region, including coal-mining activity, which may partly contextualise this spatial pattern in historically industrial regions [71]. Nevertheless, the potential well-being and social support provided by such venues should be acknowledged. Nightclubs, venues associated with heavier alcohol consumption and risky behaviours [72,73], were concentrated in Newport and Caerphilly. However, interpretation is limited by the small number of nightclubs in Gwent and the urban character of Newport.

### 4.5. Limitations

Despite its contributions, this research has several limitations that should be considered when interpreting the findings. Firstly, a key limitation of this exploratory research is the reliance on aggregate-level data throughout. As this was ecological cross-sectional research using aggregated data, this introduces the potential for ecological bias, as associations observed at the local authority level may not reflect individual-level relationships. Consequently, the exploratory findings of this research should be interpreted with caution, and individual-level conclusions cannot be inferred from group-level statistics.

The aggregate nature of the data also limited the types of analyses that could be conducted. While correlations, descriptive statistics, and geographical patterns can identify patterns and associations, they are insufficient to determine the true drivers of the alcohol-harm paradox. Numerous measures, including alcohol-specific death and alcohol-attributable hospital admission rates, were based on only five data points. Consequently, analyses involving these outcomes should be interpreted cautiously as the limited number of observations reduced the stability and reliability of results. As a result, the findings should be viewed as exploratory and interpreted as potential hypothesised contributors to the paradox rather than causal explanations. Access to individual-level data and larger sample sizes would not only reduce concerns regarding ecological fallacy but would also enable more methodologically rigorous analytical approaches to be conducted, more stable parameter estimation, and greater control for potential confounding variables.

Additionally, this research focused on a single region in South-East Wales, limiting the extent to which the findings can be generalised to other geographical contexts. The research does not provide definitive evidence regarding the causes of the alcohol-harm paradox; rather, it identifies and explores a range of potential hypotheses that warrant further exploration in other settings.

## 5. Conclusions

This research explored numerous proposed hypotheses for the alcohol-harm paradox in Gwent, South-East Wales, using an integrated exploratory analysis of demographics, health behaviours, and the geography of alcohol-related venues. The findings in Section 3 provide suggestions relevant to the research questions outlined in Section 1 and are summarised below:

1. Is there evidence of the alcohol-harm paradox in Gwent?
  - The findings are consistent with the presence of the alcohol-harm paradox in Gwent, where more deprived areas experience higher rates of alcohol-related harms despite reporting lower average annual alcohol consumption.
2. What factors are associated with the alcohol-harm paradox in Gwent?
  - Patterns of health behaviours varied across local authorities in Gwent, and were associated with differences in rates of alcohol-related harms.
  - The alcohol venue landscape differed by deprivation, suggesting that venue type, rather than density alone, may be relevant to patterns of alcohol-related harm.
  - Geographical analyses indicated that more deprived areas in Gwent were consistently closer to all types of alcohol-related venues explored, with particularly marked differences in proximity to convenience stores, social clubs and nightclubs.
  - Overall, the findings suggest that the alcohol-harm paradox is associated with a range of behavioural, socioeconomic and environmental components, rather than a single observed factor.

### Future Work

3. How can the findings be applied beyond Gwent?
  - Future research should replicate this integrated approach in other regions to assess whether similar patterns are observed elsewhere and evaluate the generalisability of the findings. By using open-access data, the research can be extended to other areas, in particular Wales and the UK.
  - The findings may inform models of the dynamics between alcohol consumption and consequent harms. The results highlight the potential value of considering behavioural, social and environmental determinants together when modelling such dynamics.
  - The research suggests several patterns that may be relevant across public health, licensing and urban planning; nevertheless, future work is needed to determine their practical implications.
  - As the research relied on routinely collected publicly available datasets, similar exploratory analyses could be conducted in other local public health and planning contexts.
  - Future research could explore how policies addressing both individual behaviours and the wider alcohol environment could interact to support a more holistic approach to reducing alcohol-related health inequalities.

This research contributes to the literature on the alcohol-harm paradox by integrating behavioural, socioeconomic and environmental factors within a single exploratory framework. Rather than identifying explanatory mechanisms, it highlights patterns and associations that may help guide future investigations of the alcohol-harm paradox.

## Figures and Tables

**Table 2:** Average annual alcohol consumption, alcohol-specific death rate per 100k, alcohol-attributable hospital admission rate per 100k, and binge drinking prevalence by deprivation (least deprived—Monmouthshire, moderately deprived—Caerphilly and Torfaen, most deprived—Blaenau Gwent and Newport) (population-level statistics).

Factor	Least Deprived	Moderately Deprived	Most Deprived
Annual consumption	656.3	457.2	431.6
Death rate	11.6	15.6	19.1
Hospital admission rate	1547.0	2012.2	2078.7
Binge drinking prevalence	8.9%	13.8%	12.1%

**Table 3:** Health behaviour prevalence by local authority.

Health Behaviour	Type	Monmouthshire (Least Deprived)	Torfaen	Caerphilly	Newport	Blaenau Gwent (Most Deprived)
Smoking	Smoker	13.7	16.6	10.8	10.2	11.9
	Ex-smoker	27.5	31.6	24.4	32.1	33.6
	Never smoked	58.9	51.9	64.8	57.7	54.5
Diet	No fruit or veg	5.3	6.5	9.5	2.2	11.9
	1–4 portions	70.2	69.8	70.3	61.6	73.9
	>5 portion	24.5	23.7	20.2	36.2	14.2
Physical activity	<30 min	37.0	41.4	32.7	26.1	49.1
	30–149 min	13.6	11.5	18.4	13.0	7.5
	150+ min	49.4	47.1	48.9	60.9	43.4
BMI	Underweight	2.3	1.1	1.8	0.6	3.0
	Healthy weight	35.3	36.2	28.0	45.0	19.2
	Overweight	34.0	36.2	38.5	33.2	40.8
	Obese	28.4	26.4	31.7	21.1	36.9
Number of healthy behaviours	0	0.0	0.8	0.5	1.4	1.8
	1	9.1	12.8	4.7	5.9	9.8
	2	18.9	24.5	36.5	39.2	37.1
	3	34.4	36.7	39.6	27.2	35.9
	4	27.1	17.7	15.9	14.8	14.0
	5	10.6	7.4	2.8	11.6	1.4

**Table 4:** Logistic regression results—odds ratios of alcohol-harm paradox variables by deprivation (* *p* <0.05).

Alcohol-Harm Paradox Variable		Odds Ratio	CI	*p*-Value
Smoking (Never baseline)	Ex-smoker	1.367	[1.010, 1.851]	0.043 *
	Smoker	2.678	[1.759, 4.075]	0.000 *
BMI (Healthy baseline)	Underweight	2.009	[0.707, 5.705]	0.190
	Overweight	1.082	[0.763, 1.536]	0.657
	Obese	1.446	[1.009, 2.072]	0.045 *
Exercise (150+ baseline)	Less than 30	1.335	[0.999, 1.785]	0.051
	30–149	1.394	[0.901, 2.157]	0.136
Diet (5+ baseline)	No portions	2.317	[1.324, 4.056]	0.003 *
	1–4 portions	1.776	[1.268, 2.489]	0.001 *
Type (Not baseline)	Beer	1.163	[0.851, 1.589]	0.343
	Wine	0.604	[0.443, 0.825]	0.002 *
	Spirits	1.047	[0.768, 1.427]	0.772
Quantity (Not binge baseline)	Beer	1.612	[0.933, 2.786]	0.087
	Wine	1.478	[0.704, 3.101]	0.303
	Spirits	0.885	[0.290, 2.705]	0.830

**Table 5:** Percentage of alcohol-related venues by local authority in Gwent.

Venue	Local Authority					
	Monmouthshire (Least Deprived)	Torfaen	Caerphilly	Newport	Blaenau Gwent (Most Deprived)	Total
Hotels	8.1	3.4	3.4	4.2	0.9	4.5
Pubs, bars and inns	31.3	18.6	23.9	19.2	26.1	24.2
Restaurants	25.2	18.6	9.0	19.2	7.2	16.6
Nightclubs	0.0	0.0	0.0	1.0	1.8	0.5
Social clubs	3.3	10.2	9.7	5.8	7.2	6.6
On-licence	68.0	50.8	46.0	49.4	43.2	52.4
Alcoholic drinks retailers	2.8	0.0	1.1	1.6	2.7	1.8
Convenience stores	21.5	40.7	46.6	45.8	48.6	40.1
Supermarkets	4.9	6.8	5.6	3.2	5.4	4.7
Drinks manufacturers	2.8	1.7	0.7	0.0	0.0	1.0
Off-licence	32.0	49.2	54.0	50.6	56.7	47.5
Total count	246	59	268	312	111	996

## Data Availability

This research explored data from publicly available sources, which are cited and described in Section 2. All analysis and code is archived at [53]. All data is archived at [54].

## Supplementary Materials

The following supporting information can be downloaded at: https://www.mdpi.com/article/10.3390/ijerph23081058/s1. Supplementary Section S1: Alcohol-related venues—points of interest; Supplementary Section S2: WIMD domains and their respective weights; Supplementary Section S3: STROBE checklist; Supplementary Section S4: Mean age and the proportion of the population over 65 by local authority in Gwent; Supplementary Section S5: Population density by local authority in Gwent; Supplementary Section S6: Bar plot of percentage of the population of Gwent in each consumption category by sex; Supplementary Section S7: Number of alcohol-related venues by local authority and LSOA and deprivation quintile; Supplementary Section S8: Average distance to the *k* nearest alcohol-related venues by local authority and deprivation quintile.
