# Supplementary material for: A Data-Driven Exploratory Analysis of the Alcohol-Harm Paradox in South-East Wales
Source: Int J Environ Res Public Health. 2026 Aug 14;23(8):1058. doi: 10.3390/ijerph23081058 (PMC13513942; doi:10.3390/ijerph23081058)
Supplement: Supplementary file 1 [file ijerph-23-01058-s001.zip › ijerph-4254542-supplementary.pdf]

A Data-Driven Exploratory Analysis of the Alcohol-Harm Paradox in South-East Wales - Supplementary Material

Elin H. Williams, Daniel Gartner, Paul R. Harper and Geraint I. Palmer-Liyu

S1 Alcohol-related venues - points of interest

- 1 Accommodation eating and drinking
  - 01 Accommodation
    - 0006 Hotels, motels, country houses and inns
  - 02 Eating and drinking
    - 0034 Pubs, bars and inns
    - 0043 Restaurants
- 4 Sport and entertainment
  - 25 Venues, stage and screen
    - 0312 Nightclubs
    - 0314 Social clubs
- 9 Retail
  - 47 Food, drink and multi item retail
    - 0671 Alcoholic drinks including off-licences and wholesalers
    - 0699 Convenience stores and independent supermarkets
    - 0819 Supermarket chains
- 7 Manufacturing and production
  - 40 Foodstuffs
    - 0522 Alcoholic drinks

S2 WIMD domains and their respective weights

**Domain**	**Weight**
Income	22%
Employment	20%
Health	15%
Education	14%
Access to services	10%
Housing	9%
Community safety	5%
Physical environment	5%

Table S1: WIMD domains and their weights

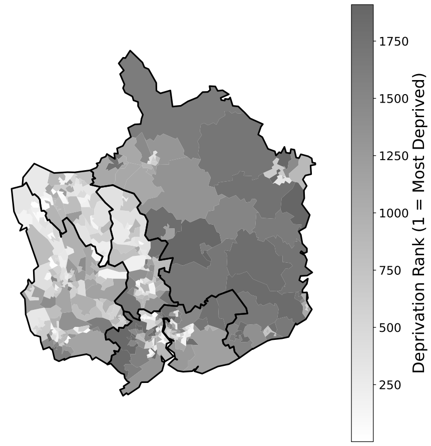

(a) Employment

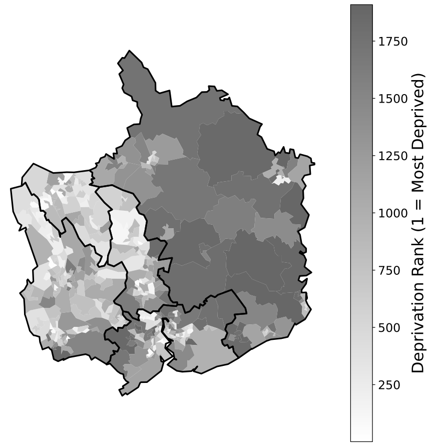

(b) Health

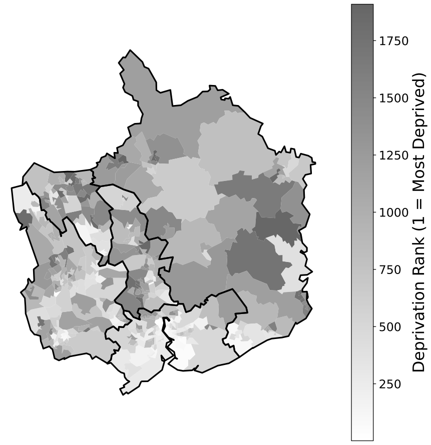

(c) Physical environment

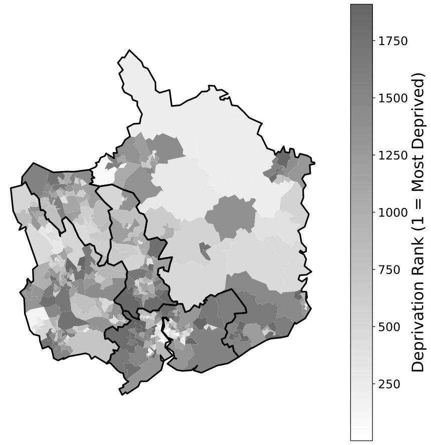

(d) Housing

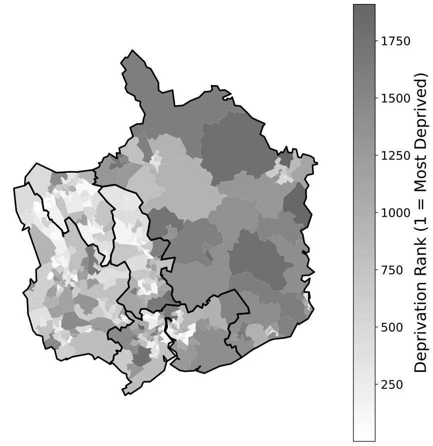

(e) Community Safety

Figure S1: Domains of WIMD across LSOAs in Gwent

S3 STROBE checklist

Item	Item number	Recommendation	Reported in section
**Title and abstract**	1	(a) Indicate the study's design with a commonly used term in the title or the abstract	Abstract
		(b) Provide in the abstract an informative and balanced summary of what was done and what was found	Abstract
**Introduction**			
Background/Rationale	2	Explain the scientific background and rationale for the investigation being reported	1
Objectives	3	State specific objectives, including any prespecified hypotheses	1
**Methods**			
Study design	4	Present key elements of study design early in the paper	2
Setting	5	Describe the setting, locations, and relevant dates, including periods of recruitment, exposure, follow-up, and data collection	2
Participants	6	Give the eligibility criteria, and the sources and methods of selection of participants	NA
Variables	7	Clearly define all outcomes, exposures, predictors, potential confounders, and effect modifiers. Give diagnostic criteria, if applicable	2
Data sources/	8	For each variable of interest, give sources of data and details of methods of assessment (measurement). Describe comparability of assessment methods if there is more than one group	2
Bias	9	Describe any efforts to address potential sources of bias	4.4
Study size	10	Explain how the study size was arrived at	2
Quantitative variables	11	Explain how quantitative variables were handled in the analyses. If applicable, describe which groupings were chosen and why	2
Statistical methods	12	(a) Describe all statistical methods, including those used to control for confounding	2
		(b) Describe any methods used to examine subgroups and interactions	3.1, 3.2, 3.3
		(c) Explain how missing data were addressed	NA
		(d) If applicable, describe analytical methods taking account of sampling strategy	2
		(e) Describe any sensitivity analyses	NA
**Results**			
Participants	13	(a) Report numbers of individuals at each stage of study—eg numbers potentially eligible, examined for eligibility, confirmed eligible, included in the study, completing follow-up, and analysed	NA

Continued on next page.

Table S2: STROBE guidelines checklist

Item	Item number	Recommendation	Reported in section
		(b) Give reasons for non-participation at each stage	NA
		(c) Consider use of a flow diagram	NA
Descriptive data	14	(a) Give characteristics of study participants (eg demographic, clinical, social) and information on exposures and potential confounders	4.4
		(b) Indicate number of participants with missing data for each variable of interest	NA
Outcome data	15	Report numbers of outcome events or summary measures	3.1
Main results	16	(a) Give unadjusted estimates and, if applicable, confounder-adjusted estimates and their precision (eg, 95% confidence interval). Make clear which confounders were adjusted for and why they were included	3.3
		(b) Report category boundaries when continuous variables were categorized	2
		(c) If relevant, consider translating estimates of relative risk into absolute risk for a meaningful time period	NA
Other analyses	17	Report other analyses done—eg analyses of subgroups and interactions, and sensitivity analyses	3.1, 3.2, 3.3
**Discussion**			
Key results	18	Summarise key results with reference to study objectives	5
Limitations	19	Discuss limitations of the study, taking into account sources of potential bias or imprecision. Discuss both direction and magnitude of any potential bias	4
Interpretation	20	Give a cautious overall interpretation of results considering objectives, limitations, multiplicity of analyses, results from similar studies, and other relevant evidence	4
Generalisability	21	Discuss the generalisability (external validity) of the study results	5
**Other information**			
Funding	22	Give the source of funding and the role of the funders for the present study and, if applicable, for the original study on which the present article is based	Funding

S4 Age

**Local authority**	**Mean age**	**Proportion 65+**
Monmouthshire (least deprived)	45.86	0.26
Torfaen	42.09	0.21
Caerphilly	42.06	0.21
Newport	39.60	0.17
Blaenau Gwent (most deprived)	42.32	0.21

Table S3: Mean age and the proportion of the population over 65 by local authority in Gwent

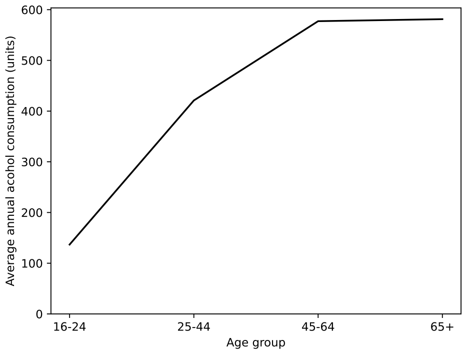

(a) Average annual alcohol consumption

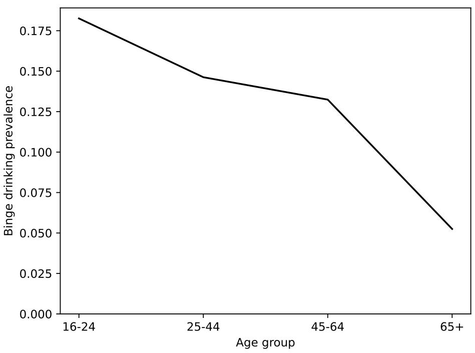

(b) Binge drinking prevalence

Figure S2: Line graphs of alcohol consumption and binge drinking by age group

S5 Urban and rural

**Local authority**	**Population density**
Monmouthshire (least deprived)	109.5
Torfaen	734.4
Caerphilly	634.2
Newport	838.1
Blaenau Gwent (most deprvied)	615.1

Table S4: Population density by local authority in Gwent [1]

S6 Sex

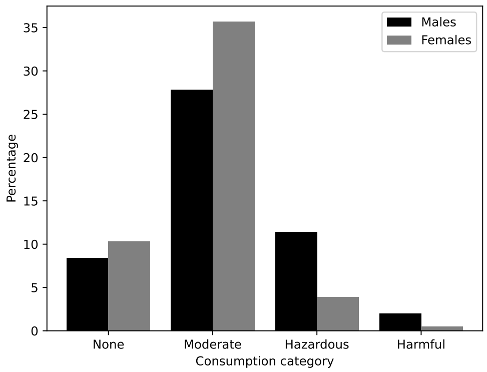

Figure S3: Bar plot of percentage of the population of Gwent in each consumption category by sex

S7 Number of alcohol-related venues by local authority and deprivation quintile

Local authority	Number of LSOAs	Venues	Number per LSOA	Population density	Area (km^2)	Number per km^2
Monmouthshire (least deprived)	58	246	4.2	109.5	849.1	0.3
Torfaen	60	59	1.0	734.4	125.7	0.5
Caerphilly	110	268	2.4	634.2	277.4	1.0
Newport	100	312	3.1	838.1	190.4	1.6
Blaenau Gwent (most deprived)	46	111	2.4	615.1	108.7	1.0

Table S5: Number of alcohol-related venues by LSOA and area

Quintile	Number of LSOAs	Venues	Number per LSOA
1	98	308	3.14
2	70	194	2.77
3	68	174	2.56
4	67	210	3.13
5	71	110	1.55

Table S6: Number of alcohol-related venues by deprivation quintile

S8 Average distance to the k nearest alcohol-related venues by local authority and deprivation quintile

Local authority	Pubs	Nightclubs	Social clubs	Convenience stores
Monmouthshire (least deprived)	3.02	24.29	11.22	3.68
Torfaen	4.06	12.85	5.77	2.33
Caerphilly	2.31	12.75	3.77	1.62
Newport	2.15	10.33	2.70	1.35
Blaenau Gwent (most deprived)	2.12	13.84	4.54	1.52

Table S7: Average distance (km) to the 10 nearest pubs, social clubs and convenience stores and five nearest nightclubs by local authority

Deprivation quintile	Pubs	Nightclubs	Social clubs	Convenience stores
1	2.43	12.50	3.90	1.49
2	2.49	13.10	4.44	1.61
3	2.47	14.47	5.02	1.90
4	2.94	15.76	6.35	2.48
5	2.91	15.07	6.06	2.56

Table S8: Average distance (km) to the 10 nearest pubs, social clubs and convenience stores, and five nearest nightclubs by deprivation quintile in Gwent
